# A uterine fibroid presenting as an incarcerated epigastric hernia: a case report and review of the literature

**DOI:** 10.1186/s13256-023-04032-7

**Published:** 2023-08-18

**Authors:** Souhaib Atri, Mahdi Hammami, Meriem Ben Brahim, Houcine Maghrebi, Montassar Kacem

**Affiliations:** Hopital la Rabta, Tunis, Tunisia

**Keywords:** Fibroids, Leiomyomas, Umbilical hernia, Epigastric hernia, Pregnancy

## Abstract

**Background:**

Uterine fibroids incarcerated in abdominal wall hernias during pregnancy are rare, with only six cases reported in umbilical hernias. This case report presents the first reported case of an incarcerated uterine fibroid in an epigastric hernia.

**Case presentation:**

A 31-year-old primigravid Caucasian woman at 28 weeks gestational age presented with sudden onset abdominal pain and vomiting. Physical examination revealed an incarcerated epigastric hernia containing a non-reducible firm mass. Ultrasound showed a healthy fetus, and during surgery, a subserosal and sessile fibroid originating from the anterior uterine wall was found in the hernia sac. It was easily reduced, and the hernia was repaired with no complications. The patient proceeded to deliver a healthy baby boy by cesarean section at full term.

**Conclusion:**

Uterine fibroids incarcerated in abdominal wall hernias during pregnancy are rare and affect mostly primigravid women in the third trimester. Abdominal ultrasound may facilitate the diagnosis, and pedunculated fibroids may be resected while sessile fibroids should be simply reduced. Clinicians should consider incarcerated fibroid as a differential diagnosis in pregnant women with irreducible ventral abdominal wall hernias. This case report aims to contribute to the literature and optimize the management of abdominal wall hernias in pregnant women.

## Background

Epigastric hernias are a protrusion of the omentum or an abdominal organ through a fascia defect in the Linea Alba above the umbilicus. The uterus is located in the pelvis and is far from the umbilicus. Due to pregnancy, the uterus is enlarged and the anterior wall can reach the umbilicus. There have been six reported cases of uterine fibroids incarcerated in an umbilical hernia during pregnancy. This is the first case of an incarcerated uterine fibroid in an epigastric hernia.

## Case presentation

A 31-year-old primigravid Caucasian woman at 28 weeks gestational age was admitted for sudden onset abdominal pain with vomiting during the last 24 h. Up to this point, she had an uncomplicated pregnancy. She reported an asymptomatic epigastric hernia that had been present for years. She had no other medical or surgical history. Physical examination showed a non-reducible epigastric firm mass indicating an incarcerated epigastric hernia. Fetal ultrasound revealed a healthy fetus with no abnormality. Given the sudden onset of her pain and our inability to reduce the hernia, she was brought emergently to the operating room with the diagnosis of an incarcerated epigastric hernia. No abdominal ultrasound was made due to the unavailability of a radiologist.

The patient received no tocolytics preoperatively due to absence of contractions and she was monitored by a gynecologist during the surgical intervention.

**Fig. 1:**
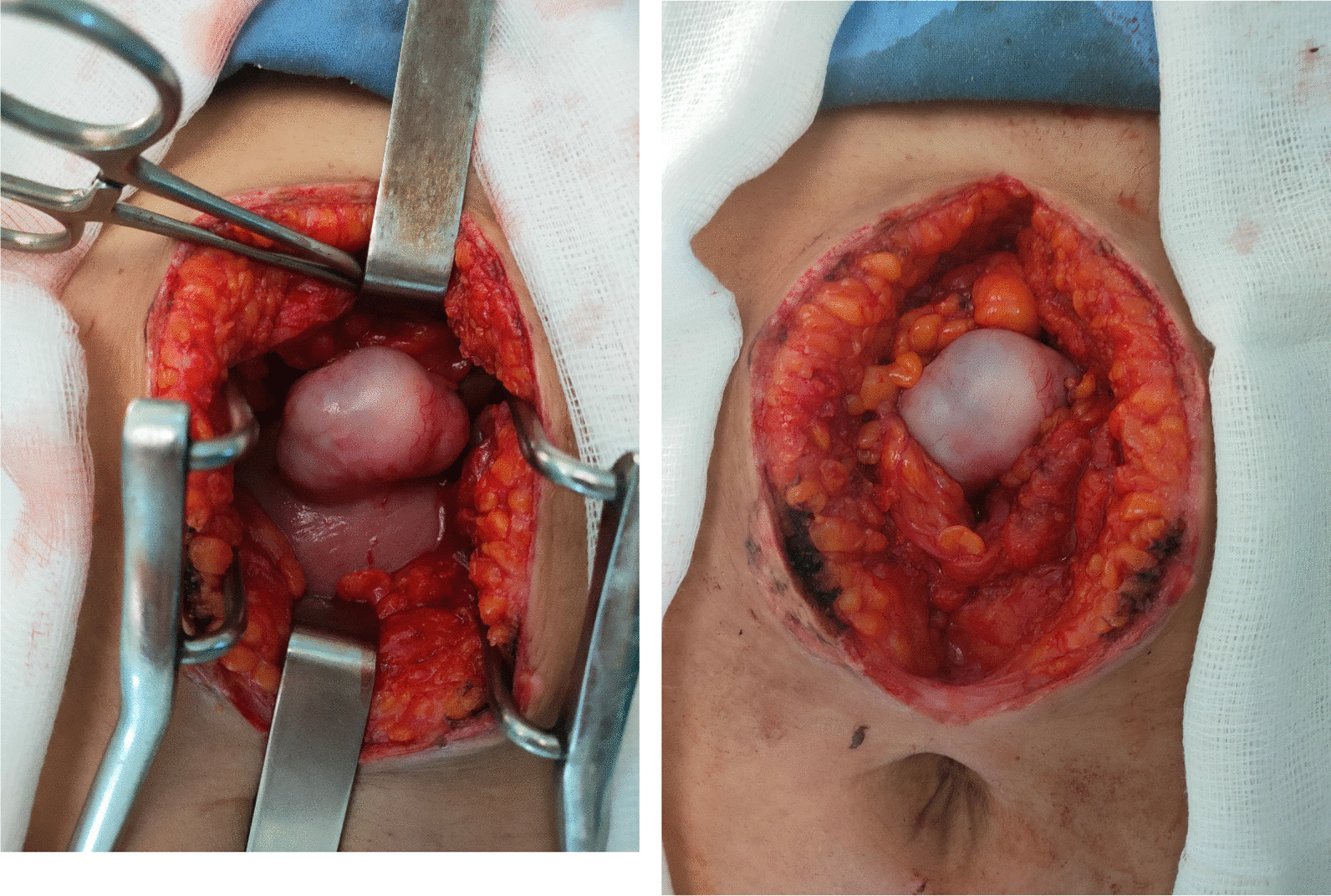
The fibroid incarcerated in the epigastric hernia

A supraumbilical incision was made under general anesthesia to perform the procedure. Upon exploration, a hernia sac measuring 3 cm in diameter was identified. Upon opening the sac, an omental fragment was discovered adhering to a sessile fibroid that originated from the anterior uterine wall (Fig. 1). It was subserosal and sessile (without a peduncle) and it was easily reduced into the abdominal cavity. As the fibroid was benign and not prone to any complication (bleeding or necrosis) no resection was performed. The hernia defect was repaired. No complications occurred in the post operative period. The patient was discharged on the third post operative day. She proceeded to a full-term pregnancy and delivered a healthy baby boy by cesarean section. The patient was examined 6 months later and showed no signs of recurring hernia.

## Discussion

The incarceration of a uterine fibroid in an umbilical or epigastric hernia is very rare and have been reported mostly in pregnant women. The uterus is typically located in the pelvis and doesn’t reach the umbilicus or the epigastric region and fibroids are typically too small to reach the umbilicus. Due to pregnancy, the uterus is enlarged and can reach the epigastric abdominal wall.

There have been six reported cases of a uterine fibroid incarcerated in an umbilical hernia during pregnancy (Table 1). All happened during the third trimester. This is the first case of a uterine fibroid incarcerated in an epigastric hernia.

**Table 1:** Cases of incarceration of a uterine fibroid in an umbilical hernia

Case no.	References	Age (yr)		Inducing factor	Symptom	Diagnosis	Size (cm)	Type	Management
1	Ehigiegba *et al*. [1]	31	Primigravid	28 weeks of gestation	Pain	Physical examination	5	Pedunculated	Myomectomy
2	Wong *et al*. [2]	30	Primigravid	32 weeks of gestation	Pain	Ultrasound	10	Pedunculated	Reduction
3	Uludag *et al*. [3]	30	Primigravid	32 weeks of gestation	Pain	Ultrasound	3	Sessile	Reduction
4	Seims *et al*. [4]	34	Primigravid	29 weeks of gestation	Pain	Physical examination	3	Sessile	Reduction
5	Kelemouridou *et al*. [5]	38	3^rd^ pregnancy	33 weeks of gestation	Pain	Physical examination	4	Pedunculated	Myomectomy
6	Zurita *et al*. [6]	39	Primigravid	22 weeks of gestation	Pain	Ultrasound	4	Pedunculated	Myomectomy
7	Current case (epigastric hernia)	31	Primigravid	28 weeks of gestation	Pain	Physical examination	3	Sessile	Reduction

The first case was reported by Ehigiegba in 1999, a 31-year-old primigravid woman at 28 weeks’ gestation presenting a pedunculated fibroid incarcerated in an umbilical hernia, which was treated by myomectomy and hernia repair.Five out of the six reported cases were primigravid pregnancies. Only one case happened in the third pregnancy. Five out of the six happened in the third trimester. Only one case happened in the second trimester. None in the first trimester.

In our case, the patient was primigravid and the incarceration of the fibroid happened in the third trimester.

Three cases were diagnosed via abdominal ultrasound. The rest relied only on physical examination, and the diagnosis of the incarcerated fibroid was made during the surgery. In our case we relied only on examination. No abdominal ultrasound was performed, which could have helped make the diagnosis pre-operatively. We suggest that every pregnant woman in the second or third trimester presenting an abdominal hernia should be explored by an abdominal ultrasound and consider an incarcerated fibroid as a differential diagnosis.

Four pedunculated fibroids were reported; three were treated by myomectomy with no further complications. The fourth case was treated by simple reduction of the fibroid and hernia repair.

Two cases of sessile fibroids were treated by hernia reduction and repair.

In our case, the fibroid was sessile with a large base of implantation so a simple reduction was performed. No myomectomy was performed due to the large base of implantation of the fibroid with a high risk of hemorrhage, miscarriage, and preterm labor.

## Conclusion

Uterine fibroid incarcerated in an abdominal wall hernia is very rare. It affects mostly primigravid pregnant women during the third trimester. Abdominal ultrasound may facilitate the diagnosis. Pedunculated fibroids may be resected while sessile fibroids should be simply reduced.The diagnosis of incarcerated fibroid should be considered as a differential diagnosis in pregnant women presenting with known uterine fibroids and an irreducible ventral abdominal wall hernia. Meticulous examination in cooperation with ultrasound evaluation are important adjunctive tools to optimize the diagnosis and decision-making toward surgical treatment.

In writing this case report, we aim to contribute to the literature by detailing what we believe to be the first reported case of fibroid incarceration in an epigastric hernia during pregnancy and to further optimize and standardize the management of abdominal wall hernias in pregnant women.

## Data Availability

Not applicable.
